# Delphinidin induces apoptosis and inhibits epithelial‐to‐mesenchymal transition via the ERK/p38 MAPK‐signaling pathway in human osteosarcoma cell lines

**DOI:** 10.1002/tox.22548

**Published:** 2018-02-16

**Authors:** Hae‐Mi Kang, Bong‐Soo Park, Hyun‐Kyung Kang, Hae‐Ryoun Park, Su‐Bin Yu, In‐Ryoung Kim

**Affiliations:** ^1^ Department of Oral Anatomy School of Dentistry, Pusan National University, Busandaehak‐ro, 49, Mulguem‐eup Yangsan‐si Gyeongsangnam‐do 50612 South Korea; ^2^ Department of Dental Hygiene Silla University, 140 Baekyang‐daero 700 beon‐gil Busan 46958 South Korea; ^3^ Department of Oral Pathology School of Dentistry, Pusan National University, Busandaehak‐ro, 49, Mulguem‐eup Yangsan‐si Gyeongsangnam‐do 50612 South Korea; ^4^ BK21 PLUS Project, School of Dentistry, Pusan National University, Busandaehak‐ro, 49, Mulguem‐eup Yangsan‐si Gyeongsangnam‐do 50612 South Korea

**Keywords:** apoptosis, delphinidin, EMT, MAPK, osteosarcoma

## Abstract

Delphinidin is major anthocyanidin that is extracted from many pigmented fruits and vegetables. This substance has anti‐oxidant, anti‐inflammatory, anti‐angiogenic, and anti‐cancer properties. In addition, delphinidin strongly suppresses the migration and invasion of various cancer cells during tumorigenesis. Although delphinidin has anti‐cancer effects, little is known about its functional roles in osteosarcoma (OS). For these reasons, we have demonstrated the effects of delphinidin on OS cell lines. The effects of delphinidin on cell viability and growth of OS cells were assessed using the MTT assay and colony formation assays. Hoechst staining indicated that the delphinidin‐treated OS cells were undergoing apoptosis. Flow cytometry, confocal microscopy, and a western blot analysis also indicated evidence of apoptosis. Inhibition of cell migration and invasion was found to be associated with epithelial‐to‐mesenchymal transition (EMT), observed by using a wound healing assay, an invasion assay, and a western blot analysis. Furthermore, delphinidin treatment resulted in a profound reduction of phosphorylated forms of ERK and p38. These findings demonstrate that delphinidin treatment suppressed EMT through the mitogen‐activated protein kinase (MAPK) signaling pathway in OS cell lines. Taken together, our results suggest that delphinidin strongly inhibits cell proliferation and induces apoptosis. Delphinidin treatment also suppresses cell migration and prevents EMT via the MAPK‐signaling pathway in OS cell lines. For these reasons, delphinidin has anti‐cancer effects and can suppress metastasis in OS cell lines, and it might be worth using as an OS therapeutic agent.

## 1 INTRODUCTION

Osteosarcoma (OS) is the most commonly observed neoplasm that causes cancer‐related deaths in children. It shares the histological finding of osteoid production in association with malignant mesenchymal cells.^1^, ^2^ OS is locally aggressive and tends to cause early systemic metastasis to non‐skeletal components, in particular, to the lungs.^3^ For these reasons, OS patients are usually treated with an aggressive chemotherapy regimen before and after surgery.^4^ However, in spite of this improvement, 20% of patients with primary metastatic OS have a 5‐year survival rate of 30%‐40%.^5^, ^6^, ^7^ Thus, it is very important to find a novel strategy for OS treatment that would effectively inhibit metastasis from the primary OS site.

Apoptosis plays a critical role in embryonic development and the elimination of damaged cells.^8^, ^9^, ^10^ It is divided into two major pathways, known as intrinsic and extrinsic pathways. The intrinsic pathway is triggered by various molecular signals, which are mitochondrial stimuli.^11^ When a problem occurs in this progression, it eventually leads to the development of cancer.^12^ For this reason, it is considered very important to kill cancer cells through various molecular mechanisms, and many anti‐cancer drugs, including natural products, are currently being studied.^13^, ^14^

Metastasis is defined as the ability of cancer cells to spread to distant organs in the body, and it accounts for more than 90% of cancer‐related deaths.^15^ These progressions are modulated by the interactions of the cell signaling pathway among the transforming growth factor‐β, the notch, the nuclear factor‐κB, wnt, and epithelial mesenchymal transition (EMT).^15^, ^16^, ^17^ EMT is a key step during embryonic morphogenesis and cancer metastasis.^18^ In aggressive cancers, EMT is characterized by decreased E‐cadherin and increased N‐cadherin expression, contributing to a stroma‐oriented adhesion with increased tumor cell motility and invasive properties in several cancers.^19^, ^20^ Additionally, several transcription factors, including the Snail/Slug family, respond to micro‐environmental stimuli and function as molecular switches for the EMT process.^21^

The mitogen‐activated protein kinase (MAPK) pathway is a key signal transmission network in eukaryotes.^22^ The MAPK family has been classified into three major subfamilies: extracellular signal‐regulated kinase (ERK), c‐Jun N‐terminal kinase (JNK/SAPK), and p38 MAPK.^23^, ^24^ These all play important roles in cell proliferation, differentiation, survival or death, migration, and invasion.^22^ In terms of metastasis, ERK, JNK, and p38 MAPK play an important role in various cancers.^25^, ^26^ ERK1/2 are serine/threonine protein kinases, and recent evidence demonstrates a specific role of ERK as a mediator of EMT in breast and colon tumors.^27^ JNK is activated by environmental and genotoxic stresses, and it has key roles in inflammation, cell proliferation, differentiation, survival, and the migration of various cancer cells.^28^, ^29^ P38 MAPK is activated by the upstream MKK3 and MKK6 kinases, and activated p38 MAPK regulates cell migration and metastasis.^30^ In addition, p38 MAPK has been implicated in the regulation of E‐cadherin and Snail and Slug protein levels.^31^

Delphinidin is a major anthocyanidin compound that is found in many pigmented fruits and vegetables,^32^ and is known to have many beneficial health effects, including anti‐oxidant,^33^ anti‐inflammatory,^34^ anti‐proliferation,^35^ anti‐cancer, and pro‐apoptotic properties.^34^, ^36^ Delphinidin strongly suppresses transformation and migration of cells during tumorigenesis in various cancers.^32^, ^37^ However, it has not been reported that delphinidin prevents cancer metastasis and induces apoptosis via the MAPK‐signaling pathway in OS.

Thus, in the present study, we demonstrate that delphinidin induces apoptosis through the mitochondria‐dependent pathway, inhibits cell motility through the MAPK‐signaling pathway, and regulates the expression of EMT‐related factors in OS cell lines. Taken together, these findings suggest that the delphinidin is valuable as a therapy for OS patients, and further research is needed.

## 2 MATERIALS AND METHODS

### 2.1 Drugs and reagents

Delphinidin (Cayman Chemical, Michigan) was dissolved in dimethyl sulfoxide (DMSO) and 10 mM delphinidin stock solution was kept at −20°C. Delphinidin stock solutions were dissolved into the culture medium as needed. Antibodies specific for caspase 3, PARP, ERK1/2, p‐ERK1/2, p38, p‐p38, JNK, and p‐JNK were purchased from Cell Signaling Technology (Beverly, Massachusetts), and antibodies against Bcl‐2, Bak, and β‐actin were obtained from Santa Cruz Biotechnology (Santa Cruz, California). Unless otherwise specified, all chemicals and laboratory supplies were obtained from Sigma Chemical Company (St. Louis, Missouri).

### 2.2 Cell culture and treatment

The human OS cell lines HOS and MG‐63 were purchased from the American Type Culture Collection (Rockville, Maryland), and U2OS was purchased from the Korean Cell Line Bank (Seoul, Korea). HOS and U2OS were cultured in RPMI 1640 media, and MG‐63 was cultured in Dulbecco's Modified Eagle Medium (DMEM) containing 10% FBS (GIBCO‐BRL, Rockville, Maryland) and 1% penicillin–streptomycin (GIBCO‐BRL, Rockville, Maryland). The cells were incubated at 37°C in a humidified atmosphere containing a 5% CO_2_ chamber. After the cells had reached 80% confluence, the culture medium was replaced with a fresh medium that was supplemented with delphinidin.

### 2.3 Cell viability assay

1 × 10^4^ cells were seeded in a 96‐well culture plate, and all of the experiments were treated with different concentrations of delphinidin (0–100 μM). After 24 h, the existing medium was removed and 100 μL of MTT (3‐[4,5‐dimethythiazol‐2‐yl]‐2,5‐diphenyltetrazolium bromide) solution (500 μg/mL) was added to each well. The cells were incubated for 4 h at 37°C. After 10 min of shaking in the SH30 orbital‐shaker, the insoluble purple formazan product was dissolved in DMSO, and absorbance of each well was measured using an ELISA reader (Tecan, Mänedorf, Switzerland) at an excitatory emission wavelength of 620 nm.

### 2.4 Colony‐forming assay

The cells were seeded into six‐well plates (3 × 10^2^ cells/well) and incubated overnight. The cells were treated with various concentrations of delphinidin. After treatment, the cells were washed and allowed to grow for 10 days until colonies formed. Colonies were fixed with 100% methanol and stained with crystal violet for 10 min at room temperature. Next, after washing with sterile distilled water, they were dried at room temperature. Clones with >50 cells were counted with an ordinary optical microscope.

### 2.5 Hoechst 33342 staining

HOS and U2OS cells were seeded into a 60 mm culture dish, treated with various concentrations of delphinidin, and incubated for 24 h. Next, cells were harvested using trypsinization and centrifuged at 3000 rpm for 5 min onto a clean glass slide. After this, the cells were stained with 1 μg/mL Hoechst 33342 for 10 min at 37°C. Following incubation, the cells were washed using phosphate buffered saline (PBS). Finally, the slides were mounted with glycerol. Each sample was observed under an epifluorescence microscope (Axioskop, Carl Zeiss, Göettingen, Germany).

### 2.6 Flow cytometry analysis

70%–80% confluent cells were cultured in 60 mm culture dishes for 24 h, and then the cells were exposed to delphinidin for 24 h. Next, the cells were harvested using trypsinization, centrifuged at 3000 rpm for 5 min, fixed with ice‐cold 70% ethanol, and stored overnight at 4°C. The next day, the fixed cells were washed in 1% bovine serum albumin in PBS solution and re‐suspended in a staining buffer of 1 mg/mL PI and 50 mg/mL RNase A, and then incubated at 4°C for 30 min. The cells were then stained with 50 μg/mL propidium iodide. Finally, the stained cells were measured using a CYTOMICS FC500 flow cytometer system (Beckman Coulter, California), and the fractions in the G1 cell cycle phase were measured for statistical analysis using the flow cytometry system. The data were analyzed using Multi Cycle software.

### 2.7 Immunofluorescence assay

HOS and U2OS cells were plated in incomplete medium in an 8‐well Lab‐Tek II Chambered Coverglas (Invitrogen, Thermo Fisher Scientific, Waltham, Massachusetts) at 3 × 10^4^ cells per chamber, incubated for 24 h, and treated with delphinidin. After 24 h, the cells were washed with PBS and stained with 100 nM MitoTracker Red at 37°C for 30 min. After washing with PBS, the cells were fixed at 4% PFA for 15 min in room temperature. Fixed cells were washed two times with PBS and permeabilized with Triton X‐100 in PBS. The cells were blocked with 1% BSA for 30 min in a shaker, and then incubated with primary antibodies in 1% BSA overnight at 4°C. The next day, the cells were washed with PBS three times for 10 min, and then incubated with fluorescein isothiocyanate‐conjugated secondary antibodies (Alexa 488) in 1% BSA‐PBS for 2 h. The slides were sealed with DAPI and immediately observed with a confocal microscope LSM700 (Cal Zeiss, Göettingen, Germany).

### 2.8 Wound healing assay (scratch assay)

HOS and U2OS cells were seeded with the same number of cells (8 × 10^4^ cells/well) in 6‐well culture plates and then incubated for 24 h. When the cells grew to 95% confluence, each well was manually scratched with 200 μL sterile pipette tips. All the wells were removed the suspended cell and washed with PBS, and then the plates were washed and incubated with delphinidin at 37°C. Images were obtained again after 24 h of incubation. The distance between two cell edges was analyzed using ImageJ software.

### 2.9 Invasion assay

The Transwell assay was done using chambers with an 8.0 μm pore polycarbonate membrane (Corning Costar, Cambridge, Massachusetts). Matrigel was coated with 40 μL on the transwell and incubated for 24 h. The next day, the cells were seeded (1.5 × 10^5^ cells in 200 μL) into the upper chamber of the transwell in a serum‐free medium. The lower chamber was filled with 10% fetal bovine serum medium (750 mL). After a 24‐h incubation at 37°C in a humidified 5% CO_2_ atmosphere, the cells were fixed in methanol and stained with hematoxylin–eosin for 15 min. The cells that invaded through the pores to the lower surface of the filter were counted under an inverted microscope (Olympus, Tokyo, Japan).

### 2.10 Western blot analysis

Cell lysates were prepared by extracting proteins with lysis buffer (pH 7.6, 50 mM Tris‐Cl, 300 mM NaCl, 0.5% Triton X‐100, 2 μL/mL aprotinin, 2 mM PMSF, and 2 μL/mL leupeptin). The lysate samples were centrifuged at a 13 200 rpm for 30 min at 4°C. Protein amounts were measured using a Bradford protein assay (Bio‐Rad, Richmond, California). Cell lysates were then exposed to sodium dodecyl sulfate‐polyacrylamide gel electrophoresis (PAGE) and transferred onto a polyvinyl difluoride membrane using a wet transfer apparatus. Next, the membranes were blocked with a 5% non‐fat dry milk blocking buffer for 2 h at room temperature. After blocking, the membranes were kept at 4°C for 24 h with the respective primary antibodies. The membranes were washed for 1 h and treated with secondary antibodies for 2 h at room temperature. Finally, the membranes were washed again for 1 h, and detection of each protein was performed using a Super Signal West Femto (Pierce, Rockford, Illinois) and analyzed using an Alpha Imager HP (Alpha Innotech, Santa Clara).

### 2.11 Statistical analysis

Data were expressed as mean ± SEM. Statistical analyses were performed with the student's *t* test for comparing treatment values and control values, using GraphPad Prism (GraphPad Software, San Diego, California). A one‐way ANOVA was used for Dunnett's multiple‐comparison test in the statistical analysis.

## 3 RESULTS

### 3.1 Delphinidin reduces cell viability and proliferation of OS cell lines

**Figure 1:**
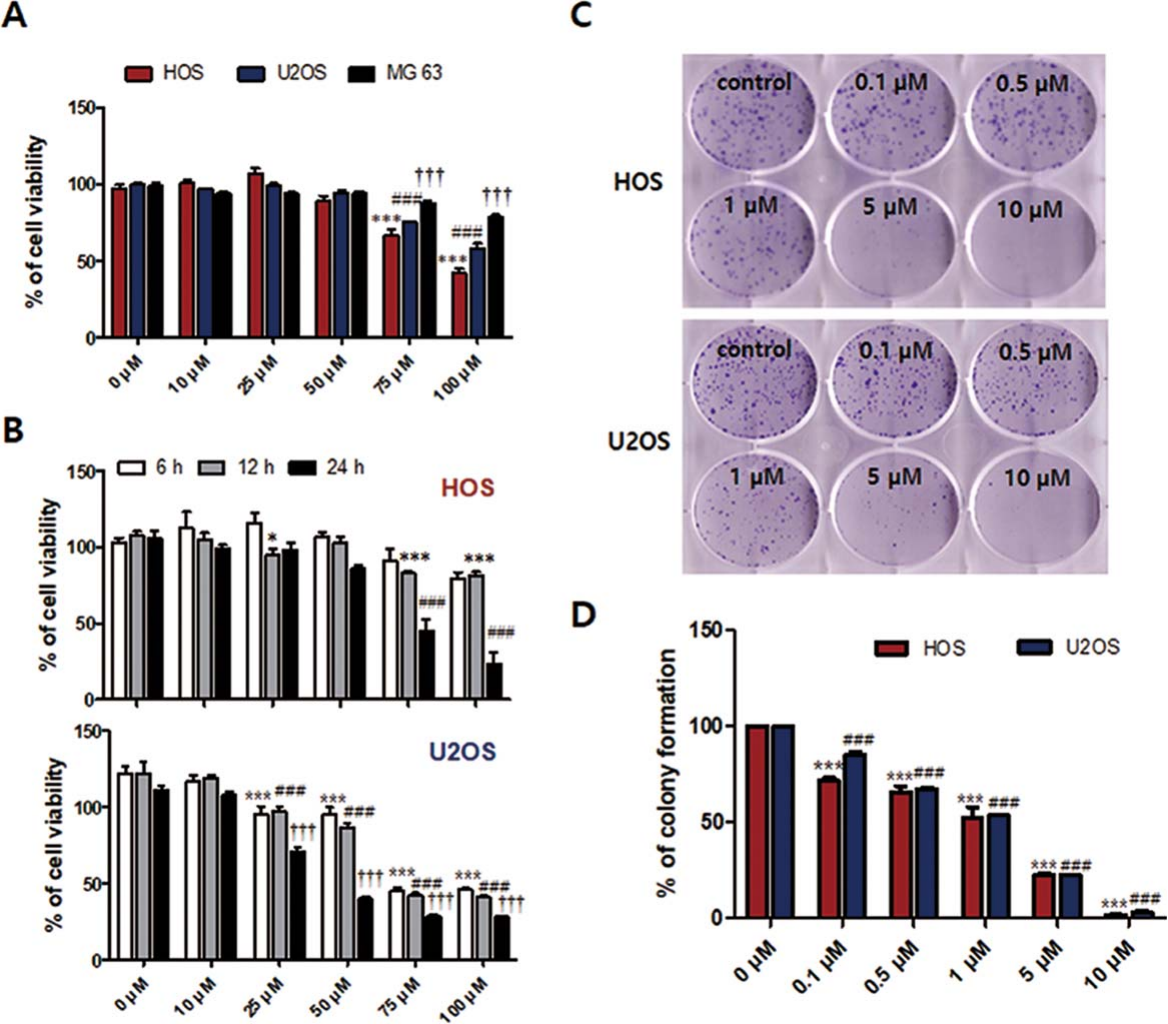
Delphinidin reduced cell viability and cell proliferation in OS cell lines. (A) OS cell lines (HOS, U2OS, and MG‐63) were treated with delphinidin (0–100 μM) for 24 h and measured using the MTT assay. The data are expressed as the mean ± SEM (*n* = 6). The statistical significance of each group was analyzed by a one‐way ANOVA and Dunnett test [***P < 0.001 at HOS, ###*P* < 0.001 at U2OS, and †††*P* < 0.001 at MG63 for 24 h compared to the non‐treated group (0 μM)]. (B) HOS and U2OS cells were treated with 0–100 µM of delphinidin for 6–24 h (*P < 0.05, ***P < 0.001 at 12 h and ###*P* < 0.001 at 24 h compared to the non‐treated group (0 μM) in HOS cell; ***P < 0.001 at 6 h, ###*P* < 0.001 at 12 h and †††*P* < 0.001 at 24 h compared to the non‐treated group (0 μM) in U2OS cell). (C) HOS and U2OS cells were treated with various concentrations (0.1–10 µM) of delphinidin for 7 days, and were observed using a colony‐forming assay. (D) Quantification of the colony number described in C The data are expressed as the mean ± SEM (*n* = 3) [***P < 0.001 at HOS and ###*P* < 0.001 at U2OS for 7 days compared to the control group (0 μM)] [Color figure can be viewed at http://wileyonlinelibrary.com]

To confirm the effect of delphinidin on the cell viability of OS cell lines, 0–100 μM on HOS, MG‐63, and U2OS cells were treated with delphinidin for 24 h. As shown in Figure 1A, delphinidin decreased the cell viability of HOS and U2OS cells in a dose‐dependent manner, but in MG‐63 cells, delphinidin showed minimal cell damage. Based on these results, we selected HOS and U2OS cells and checked cell viability in different time conditions (6–24 h) of delphinidin. As a result, cell viability decreased dose‐ and time‐dependently in both cell lines (Figure 1B). To observe the effect of delphinidin on proliferation of HOS and U2OS, we conducted a colony‐forming assay. As shown in Figure 1C, delphinidin dramatically inhibited the proliferation of HOS and U2OS cells at a low dose. It is shown in the histograms (Figure 1D) that delphinidin inhibits cell proliferation on both cell lines. These results indicate the delphinidin treatment reduced cell viability and inhibited cell proliferation in OS cell lines.

### 3.2 Delphinidin induces apoptosis via the mitochondrial‐mediated pathway in OS cells

**Figure 2:**
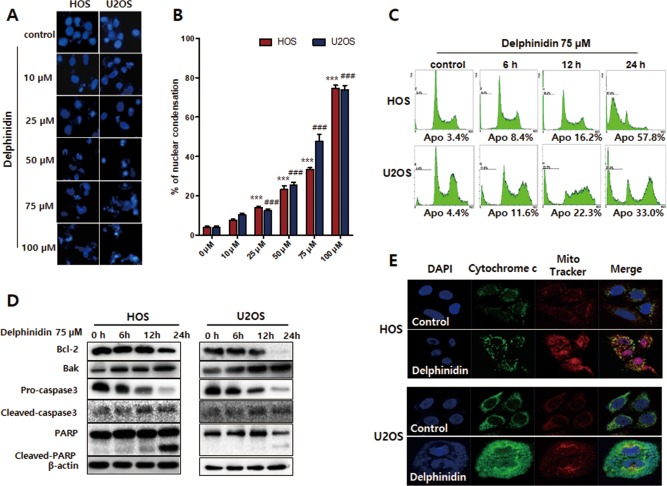
Delphinidin induced morphological changes and apoptosis through the mitochondrial‐mediated pathway in HOS and U2OS cells. (A) Apoptotic nuclei manifested condensed and fragmented DNA, brightly stained by Hoechst staining. (B) The percentage of apoptosis cells was calculated and shown in histograms. The data are expressed as the mean ± SEM (*n* = 3). (***P < 0.001 at HOS and ###*P* < 0.001 at U2OS) (C) The apoptosis ratio of HOS and U2OS was measured using flow cytometry. (D) HOS and U2OS cells were treated with 75 µM delphinidin for 6–24 h and then examined using a western blot analysis. (E) Emission of the cytochrome *c* from the mitochondria into the cytosol was analyzed with a confocal microscope [Color figure can be viewed at http://wileyonlinelibrary.com]

Hoechst staining was used to assess nucleic morphological changes in HOS and U2OS cells using a fluorescence microscope. As shown in Figure 2A, the control group has the round shape of nuclei and it was stained homogeneously blue; on the other hand, the delphinidin treatment groups (10–100 μM) were excess significantly nuclear condense and shrinkage in the both cells. The nuclei condensation ratio showed a marked, dose‐dependent increase. In particular, the 75 μM delphinidin treatment group showed a nuclear condensation ratio of 37% and 48% in HOS and U2OS (Figure 2B). In the next experiment, the delphinidin 75 μM group was treated for 6–24 h in HOS and U2OS cells, and its apoptotic ratio was conducted using flow cytometry. The apoptotic ratio dramatically increased in a time‐dependent manner with 75 μM delphinidin treatment, at 3.4% (0 h), 8.4% (6 h), 16.2% (12 h), and 57.8% (24 h) in HOS, and 4.4% (0 h), 11.6% (6 h), 22.3% (12 h), and 33% (24 h) in U2OS (Figure 2C).

To determine the molecular mechanism of apoptosis with delphinidin treatment in HOS and U2OS cells, the apoptosis‐related proteins were assessed using a western blot analysis. Delphinidin treatment in HOS and U2OS cells showed that the anti‐apoptotic protein Bcl‐2 was down‐regulated, and the pro‐apoptotic protein Bak was up‐regulated in a time‐dependent manner. Additionally, pro‐caspase‐3, cleavage caspase‐3, and PARP were activated, and triggered the release of cytochrome *c* from the mitochondria to the cytosol in both cell lines (Figure 2D‐F). Overall, these results suggest that delphinidin‐induced apoptosis occurs via a mitochondrial‐dependent pathway.

### 3.3 Delphinidin to inhibit cell invasion capacities and modulate the expression of EMT markers

**Figure 3:**
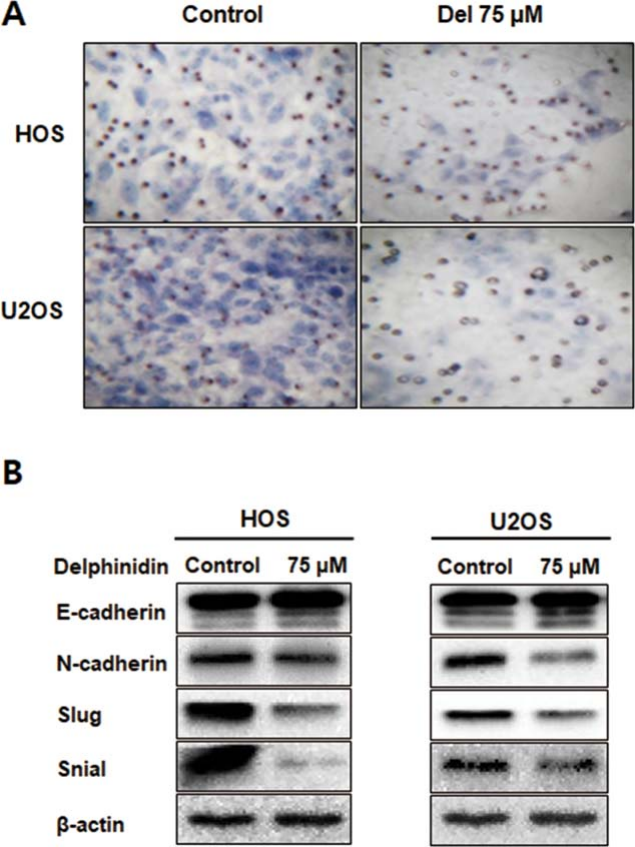
Delphinidin inhibited OS cell invasion and regulated the expression of EMT markers. (A) Transwell assay was employed to examine the invasion ability of the delphinidin‐treated OS cells. (B) The expression of EMT markers was detected using a western blot analysis. The levels of β‐actin were used as an internal control [Color figure can be viewed at http://wileyonlinelibrary.com]

To further examine the effect of delphinidin on HOS and U2OS cell invasion, we used matrigel‐coated transwell chambers, and both cells were treated with 75 μM delphinidin for 24 h. Invasive cells were significantly inhibited in the delphinidin treatment groups in both types of cells (Figure 3A). Western blot results showed that the delphinidin treatment up‐regulated the expression of epithelial markers such as E‐cadherin. On the other hand, the mesenchymal marker N‐cadherin was down‐regulated with delphinidin treatment. The transcription factors of the Snail and Slug expression levels were significantly decreased in the delphinidin treatment group (Figure 3B). These results indicate that delphinidin inhibits cell invasion and modulates the expression of EMT‐related markers of OS cells.

### 3.4 Delphinidin to inhibit the migration of OS cell lines via the MAPK‐signaling pathway

**Figure 4:**
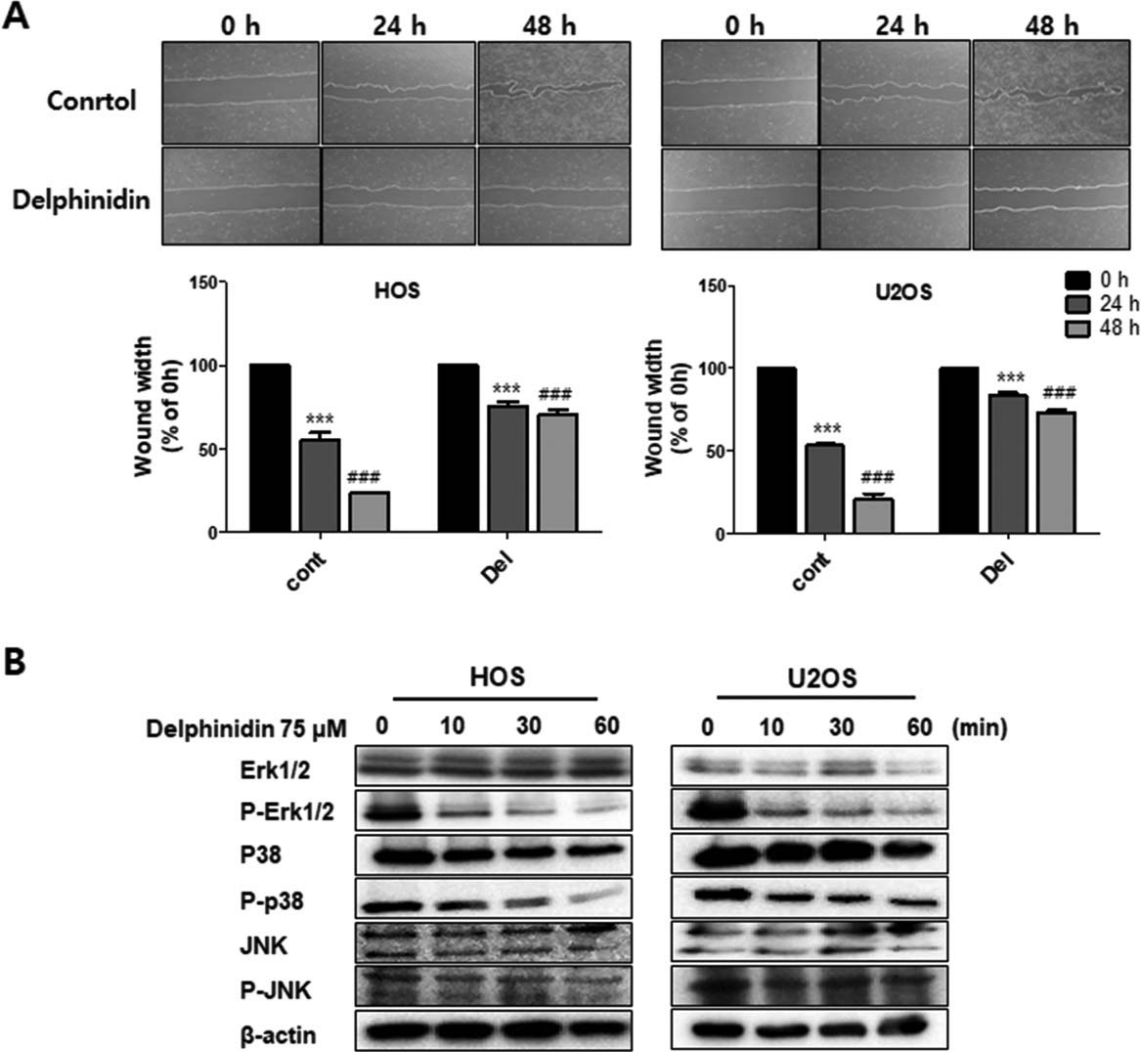
Delphinidin inhibits cell migration via the MAPK‐signaling pathway. (A) Cell migration was determined using a wound healing assay after 24 h of treatment with 75 μM delphinidin. The data are expressed as the mean ± SEM (*n* = 3). The statistical significance of each group was analyzed by a two‐way ANOVA test [***P < 0.001 at HOS and ###*P* < 0.001 at U2OS compared to the non‐treated group (0 μM)]. (B) The MAPK‐signaling pathway‐related protein levels were assayed with a western blot. β‐actin was used as an internal control

To investigate the effect of delphinidin on HOS and U2OS cell migration, we performed the wound healing assay. In the delphinidin 75 μM treatment group, migration width was inhibited compared to the untreated cells in both cell lines. To examine the association between delphinidin and the MAPK family (ERK1/2, p38, and JNK), we checked its protein expression using a western blot analysis. The expression levels of p‐ERK1/2 and p‐p38 in both cells were down‐regulated time‐dependently whereas p‐JNK remained unchanged compared to the 0 min control (Figure 4B). These findings suggest that delphinidin treatment can markedly suppress cell motility and regulate the MAPK‐signaling pathway in OS cells.

### 3.5 Inhibition of the MAPK‐signaling pathway to suppress the EMT effect of delphinidin in OS cells

**Figure 5:**
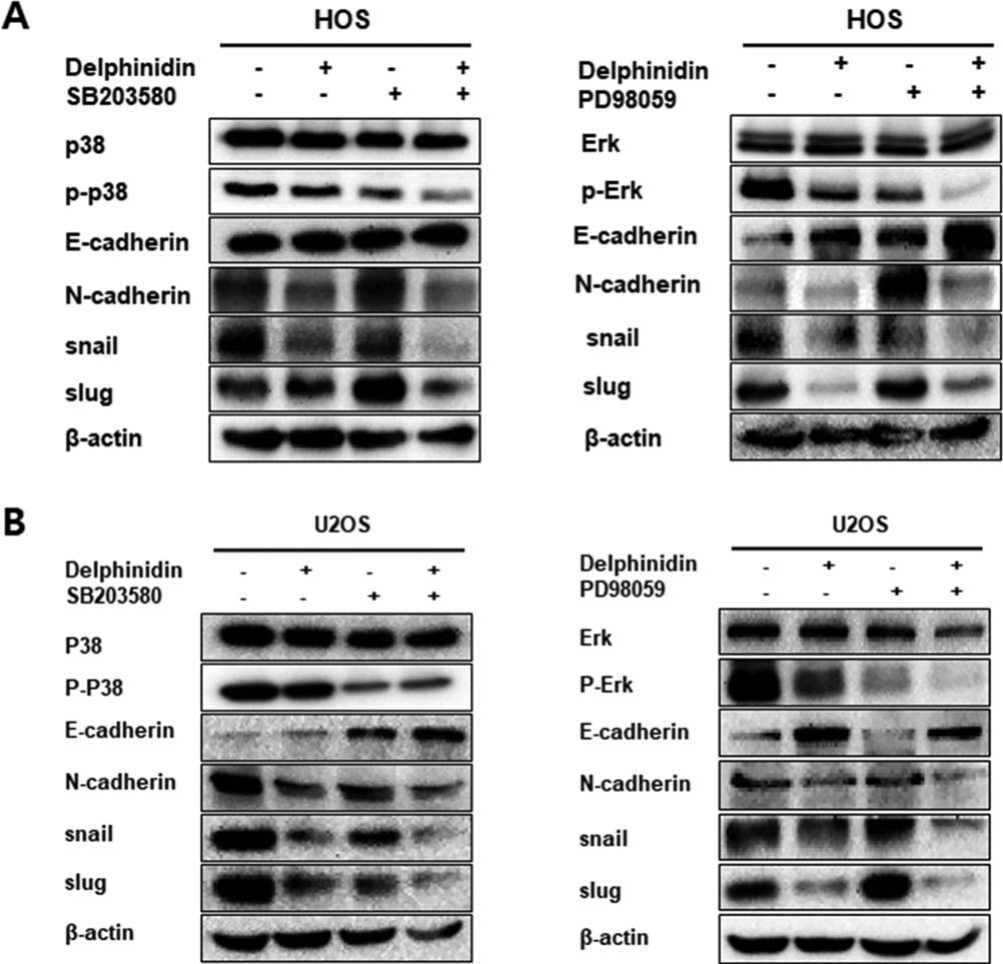
Inhibition of ERK and p38 suppressed the EMT effect of delphinidin in OS cells. (A,B) The OS cells were pretreated with an ERK1/2 and p38 inhibitor, and were detected using a western blot analysis. The levels of β‐actin were used as an internal standard for quantifying ERK, p38, and EMT factor expression

In order to further investigate whether the inhibition of delphinidin was mainly through inhibition of the ERK1/2 and p38 MAPK‐signaling pathways, we used an ERK1/2 and p38 inhibitor (SB203580 and PD38059, 20 μM). Inhibition of ERK1/2 and p38 using a SB203580 and PD38059 were to enhance the expression of E‐cadherin compared with delphinidin treatment alone in HOS and U2OS cell lines. The expression of the mesenchymal marker N‐cadherin and the transcription factors of the Snail and Slug expression levels was significantly reduced compared to delphinidin treatment alone (Figure 5A,B). Therefore, the present study supports the hypothesis that inhibition of metastasis in OS cells with delphinidin is modulated by suppression via the MAPK‐signaling pathway.

## 4 DISCUSSION

Metastasis is a very important process in human cancers.^38^, ^39^ This progression occurs when unstable cancer cells migrate to a tissue microenvironment that is distant from the primary tumor.^40^ Cancer cells need to be able to migrate and invade surrounding local tissue, which usually involves a process referred to as EMT.^31^ They then enter the microvasculature of the lymph and blood systems and translocate that through the bloodstream to the micro‐vessels of distant tissues. Finally, they adapt to the foreign microenvironment of these tissues in ways that facilitate cell proliferation and the formation of a macroscopic secondary tumor.^41^

OS is a malignant tumor that is observed mainly in children and is a highly aggressive tumor that metastasizes primarily to the lungs.^42^ Thus, we have conducted various experiments to investigate the effect of delphinidin, which can inhibit OS metastasis. In other studies, delphinidin inhibits HER‐2 and ERK1/2 signaling and induces apoptosis in breast cancer cell lines.^32^ Furthermore, delphinidin suppresses proliferation and migration of human ovarian clear cell carcinoma cells.^43^ In the present study, we performed a MTT assay to compare the cytotoxic effects of delphinidin treatment on OS cell lines (HOS, U2OS, and MG‐63 cells). Delphinidin treatment was shown to decrease the cell viability of HOS and U2OS cells dose‐ and time‐dependently, but the MG‐63 cell showed little effect from delphinidin treatment. Therefore, we conducted experiments using HOS and U2OS cells, which showed the cytotoxic effects of delphinidin. In order to confirm the cell proliferation effect of delphinidin, a very low concentration (0.1–10 μM) of delphinidin was used, and then a colony‐forming assay was performed. As a result, delphinidin inhibited the proliferation of HOS and U2OS cells; in particular, a dose higher than 1 μM of delphinidin showed a sharp decrease. Based on these results, we found that delphinidin has a cytotoxicity effect in OS cell lines and that it inhibited cell proliferation at very low concentrations. When apoptosis progresses, the nucleus of the cell undergoes a morphological change.^44^ These changes almost invariably involve chromatin condensation, DNA fragmentation, and cellular shrinkage and blebbing.^44^, ^45^ In another published study, delphinidin‐induced DNA fragmentation and morphological changes in human ovarian clear cell carcinoma cells.^43^ To evaluate the morphological changes of nuclei during the apoptosis progression with delphinidin, we performed Hoechst 33342 staining. As a result, delphinidin‐treated OS cells revealed apoptotic hallmarks such as DNA fragmentation and formation of apoptotic bodies. On the other hand, the control cells did not reveal apoptotic bodies. These results indicate that delphinidin treatment definitely induces morphological changes and nuclear condensation in OS cells. In addition, flow cytometry analysis was used to observe changes in the apoptosis ratio with delphinidin. Delphinidin‐treated OS cells had a time‐dependent increase in the apoptosis ratio. In the apoptosis process, there are two main apoptotic pathways called the intrinsic and extrinsic pathways.^46^ In the intrinsic pathway, mitochondrial dysfunction is an important key event of apoptosis that plays a vital role in the execution of both extrinsic and intrinsic apoptotic pathways.^47^ Mitochondrial membrane depolarization is an early event of the apoptosis process, and a loss of the mitochondrial membrane potentially leads to the release of the pro‐apoptotic protein Bax.^48^ The cytochrome *c* in cytosol released by Bax interacts with the Apaf‐1 and pro‐caspase‐9 complex and activates the down‐regulation of the caspase cascade.^48^, ^49^ Our results show a significant shift in the ratio of Bcl‐2 and Bak expression, the anti‐apoptotic protein Bcl‐2 decreased, and the pro‐apoptotic protein Bak increased time‐dependently with delphinidin treatment. Additionally, the expression levels of pro‐caspase‐3, cleavage caspase‐3, and PARP caused significant time‐dependent increases in OS cells. To observe the emission of cytochrome *c* from the mitochondria into the cytosol in OS cells, we used a confocal microscope. These findings clearly suggest delphinidin‐induced apoptosis occurs via the mitochondrial‐signaling pathway.

The EMT process is regulated by E‐cadherin (epithelial marker), N‐cadherin (mesenchymal marker), and various transcription factors.^31^ The master regulator, E‐cadherin, is related to cell–cell adhesion, and the connection loss of E‐cadherin in cancer cells which increases the tumor grade, metastasis, and mortality of various cancer cells.^50^, ^51^ Well‐known EMT‐related transcription factor, such as Snail, Slug, Twist, ZEB1, and ZEB2 respond to microenvironmental stimuli and function as molecular switches for the EMT process.^52^ Snail is to encode a zinc‐finger transcriptional repressor controlling EMT during tumor progression.^53^ Snail genes have subdivided into two subfamilies as Snail and Slug.^54^ Recent evidence demonstrated that snail and slug were to affect extracellular matrix components regulation, a loss of intercellular cohesion, increased rate of cellular migration and invasion and increased resistance to apoptosis, and, it is important role in cancer metastasis.^55^, ^56^, ^57^ Also, recently Snail and Slug showed to enhance the invasion in an in vitro invasion model.^58^, ^59^, ^60^ In this study, we showed the effect of delphinidin on the metastatic ability of OS cells. The delphinidin‐treated OS cells significantly decreased cell invasion and EMT‐related transcription factors at a concentration of 75 μM (Figure 3A). The expression of the epithelial marker E‐cadherin was up‐regulated, and the expression of the mesenchymal marker N‐cadherin was down‐regulated. Moreover, the expression of the Slug and Snail transcription factors was decreased in a dose‐dependent manner with delphinidin. In conclusion, our results demonstrate that delphinidin is able to inhibit cell invasion by regulating EMT‐related protein and transcription factors.

Activation of the ERK‐signaling pathways markedly reduced cellular migration, invasion, and metastasis of cancer cells.^61^ The JNK is a master protein kinases that regulates many physiological processes, including cell proliferation, differentiation, survival, and death.^29^, ^62^ The p38 MAPK regulates cellular responses to the environment and controls gene expression, cell growth, cell migration, and invasion.^63^ Several studies have shown that an increase in the p38 MAPKs plays an important role in the formation of metastatic tumors.^64^ In this study, we performed the wound healing assay, which is the most commonly used assay for cell migration measurement. Compared to the control group, delphinidin treatment significantly inhibited cell migration in OS cell lines. As shown by the western blot analysis, ERK1/2 and p38 phosphorylation were significantly decreased with delphinidin treatment in a time‐dependent manner, but there was no effect in the expression of JNK. These findings suggest that delphinidin can markedly suppress cell motility in OS cells via the MAPK‐signaling pathway. Based on these results, we confirmed the expression of EMT‐related protein again after treatment with a MAPK inhibitor (p38 inhibitor, SB203580 and ERK inhibitor, PD38059). As expected, the expression of E‐cadherin increased more when the inhibitor was treated with delphinidin than when treated alone whereas the expression of N‐cadherin and Slug and Snail was decreased in HOS cells. The expression levels of E‐cadherin and N‐cadherin were lower in U2OS than HOS cells, however expression tendency of snail and slug were a similar with in HOS cells. Therefore, these results indicated that delphinidin suppressed the EMT via MAPK signaling pathway.

In conclusion, the present study demonstrates that delphinidin showed evidence of various types of apoptosis in OS cells, and it induced apoptosis through the mitochondrial pathway. Additionally, through the experiments on metastasis in OS cells, delphinidin was able to inhibit cell invasion and migration in OS cells. Furthermore, we found that delphinidin regulates metastasis‐related protein expression levels via the MAPK‐signaling pathway. As a result, we have demonstrated that delphinidin has the effect of inhibiting cancer cell metastasis in OS cells, and delphinidin is valuable as a therapeutic tool for OS patients.

## CONFLICT OF INTEREST

All authors declare there are no conflicts of interest.

## COMPLIANCE WITH ETHICAL STANDARDS

This article does not contain studies on animal models or human subjects.

## References

[1] Trieb K , Lehner R , Stulnig T , Sulzbacher I , Shroyer KR. Survivin expression in human osteosarcoma is a marker for survival. Eur J Surg Oncol. 2003;29(4):379–382. 12711293 10.1053/ejso.2002.1415

[2] Espey DK , Wu XC , Swan J , et al. Annual report to the nation on the status of cancer, 1975–2004, featuring cancer in American Indians and Alaska Natives. Cancer. 2007;110(10):2119–2152. 17939129 10.1002/cncr.23044

[3] Endo‐Munoz L , Evdokiou A , Saunders NA. The role of osteoclasts and tumour‐associated macrophages in osteosarcoma metastasis. Biochim Biophys Acta. 2012;1826(2):434–442. 22846337 10.1016/j.bbcan.2012.07.003

[4] Raymond AK , Jaffe N. Osteosarcoma multidisciplinary approach to the management from the pathologist's perspective. Cancer Treat Res. 2009;152:63–84. 20213386 10.1007/978-1-4419-0284-9_4

[5] Desandes E. Survival from adolescent cancer. Cancer Treat Rev. 2007;33(7):609–615. 17398011 10.1016/j.ctrv.2006.12.007

[6] Jiang Z , Chen X , Chen K , et al. YAP Inhibition by resveratrol via activation of AMPK enhances the sensitivity of pancreatic cancer cells to gemcitabine. Nutrients. 2016;8(12):546 10.3390/nu8100546 PMC5083973 27669292

[7] Mialou V , Philip T , Kalifa C , et al. Metastatic osteosarcoma at diagnosis ‐ prognostic factors and long‐term outcome ‐ The French pediatric experience. Cancer. 2005;104(5):1100–1109. 16015627 10.1002/cncr.21263

[8] Chinkwo KA. Sutherlandia frutescens extracts can induce apoptosis in cultured carcinoma cells. J Ethnopharmacol. 2005;98(1–2):163–170. 15763378 10.1016/j.jep.2005.01.016

[9] Kroemer G , Martin SJ. Caspase‐independent cell death. Nat Med. 2005;11(7):725–730. 16015365 10.1038/nm1263

[10] Furth PA. Introduction: mammary gland involution and apoptosis of mammary epithelial cells. J Mammary Gland Biol Neoplasia. 1999;4(2):123–127. 10426390 10.1023/a:1018764922082

[11] Mukhopadhyay S , Panda PK , Sinha N , Das DN , Bhutia SK. Autophagy and apoptosis: where do they meet?. Apoptosis. 2014;19(4):555–566. 24415198 10.1007/s10495-014-0967-2

[12] Booth LA , Tavallai S , Hamed HA , Cruickshanks N , Dent P. The role of cell signalling in the crosstalk between autophagy and apoptosis. Cell Signal. 2014;26(3):549–555. 24308968 10.1016/j.cellsig.2013.11.028 PMC4054685

[13] Ashkenazi A. Targeting the extrinsic apoptosis pathway in cancer. Cytokine Growth Factor Rev. 2008;19(3–4):325–331. 18495520 10.1016/j.cytogfr.2008.04.001

[14] Bold RJ , Termuhlen PM , McConkey DJ. Apoptosis, cancer and cancer therapy. Surg Oncol. 1997;6(3):133–142. 9576629 10.1016/s0960-7404(97)00015-7

[15] Spano D , Heck C , De Antonellis P , Christofori G , Zollo M. Molecular networks that regulate cancer metastasis. Semin Cancer Biol. 2012;22(3):234–249. 22484561 10.1016/j.semcancer.2012.03.006

[16] Tan DSP , Agarwal R , Kaye SB. Mechanisms of transcoelomic metastasis in ovarian cancer. Lancet Oncol. 2006;7(11):925–934. 17081918 10.1016/S1470-2045(06)70939-1

[17] Wai Wong C , Dye DE , Coombe DR. The role of immunoglobulin superfamily cell adhesion molecules in cancer metastasis. Int J Cell Biol. 2012;2012:340296 22272201 10.1155/2012/340296 PMC3261479

[18] Wu Y , Zhou BP. New insights of epithelial‐mesenchymal transition in cancer metastasis. Acta Biochim Biophys Sin (Shanghai). 2008;40(7):643–650. 18604456 10.1111/j.1745-7270.2008.00443.x PMC2740620

[19] Gravdal K , Halvorsen OJ , Haukaas SA , Akslen LA. A switch from E‐cadherin to N‐cadherin expression indicates epithelial to mesenchymal transition and is of strong and independent importance for the progress of prostate cancer. Clin Cancer Res. 2007;13(23):7003–7011. 18056176 10.1158/1078-0432.CCR-07-1263

[20] Tran NL , Nagle RB , Cress AE , Heimark RL. N‐Cadherin expression in human prostate carcinoma cell lines. An epithelial‐mesenchymal transformation mediating adhesion withStromal cells. Am J Pathol. 1999;155(3):787–798. 10487836 10.1016/S0002-9440(10)65177-2 PMC1866912

[21] Wang Y , Shi J , Chai K , Ying X , Zhou BP. The role of snail in EMT and tumorigenesis. Curr Cancer Drug Targets. 2013;13(9):963–972. 24168186 10.2174/15680096113136660102 PMC4004763

[22] Chang L , Karin M. Mammalian MAP kinase signalling cascades. Nature. 2001;410(6824):37–40. 11242034 10.1038/35065000

[23] Davis RJ. Signal transduction by the JNK group of MAP kinases. Cell. 2000;103(2):239–252. 11057897 10.1016/s0092-8674(00)00116-1

[24] Lee JT , McCubrey JA. The Raf/MEK/ERK signal transduction cascade as a target for chemotherapeutic intervention in leukemia. Leukemia. 2002;16(4):486–507. 11960326 10.1038/sj.leu.2402460

[25] Dong C , Davis RJ , Flavell RA. MAP kinases in the immune response. Annu Rev Immunol. 2002;20:55–72. 11861597 10.1146/annurev.immunol.20.091301.131133

[26] Park J‐I , Lee M‐G , Cho K , et al. Transforming growth factor‐beta1 activates interleukin‐6 expression in prostate cancer cells through the synergistic collaboration of the Smad2, p38‐NF‐kappaB, JNK, and Ras signaling pathways. Oncogene. 2003;22(28):4314–4332. 12853969 10.1038/sj.onc.1206478

[27] Murugan AK , Dong JL , Xie JW , Xing MZ. MEK1 mutations, but not ERK2 mutations, occur in melanomas and colon carcinomas, but none in thyroid carcinomas. Cell Cycle. 2009;8(13):2122–2124. 19411838 10.4161/cc.8.13.8710

[28] Karin M , Gallagher E. From JNK to pay dirt: Jun kinases, their biochemistry, physiology and clinical importance. Iubmb Life. 2005;57(4–5):283–295. 16036612 10.1080/15216540500097111

[29] Bubici C , Papa S. JNK signalling in cancer: in need of new, smarter therapeutic targets. Br J Pharmacol. 2014;171(1):24–37. 24117156 10.1111/bph.12432 PMC3874694

[30] Gaundar SS , Bendall LJ. The potential and limitations of p38MAPK as a drug target for the treatment of hematological malignancies. Curr Drug Targets. 2010;11(7):823–833. 20370645 10.2174/138945010791320854

[31] Thiery JP , Acloque H , Huang RY , Nieto MA. Epithelial‐mesenchymal transitions in development and disease. Cell. 2009;139(5):871–890. 19945376 10.1016/j.cell.2009.11.007

[32] Ozbay T , Nahta R. Delphinidin inhibits HER2 and Erk1/2 signaling and suppresses growth of HER2‐overexpressing and triple negative breast cancer cell lines. Breast Cancer (Auckl). 2011;5:143–154. 21792311 10.4137/BCBCR.S7156 PMC3140266

[33] Noda Y , Kaneyuki T , Mori A , Packer L. Antioxidant activities of pomegranate fruit extract and its anthocyanidins: delphinidin, cyanidin, and pelargonidin. J Agric Food Chem. 2002;50(1):166–171. 11754562 10.1021/jf0108765

[34] Hou DX , Yanagita T , Uto T , Masuzaki S , Fujii M. Anthocyanidins inhibit cyclooxygenase‐2 expression in LPS‐evoked macrophages: structure‐activity relationship and molecular mechanisms involved. Biochem Pharmacol. 2005;70(3):417–425. 15963474 10.1016/j.bcp.2005.05.003

[35] Favot L , Martin S , Keravis T , Andriantsitohaina R , Lugnier C. Involvement of cyclin‐dependent pathway in the inhibitory effect of delphinidin on angiogenesis. Cardiovasc Res. 2003;59(2):479–487. 12909331 10.1016/s0008-6363(03)00433-4

[36] Hafeez BB , Siddiqui IA , Asim M , et al. A dietary anthocyanidin delphinidin induces apoptosis of human prostate cancer PC3 cells in vitro and in vivo: involvement of nuclear factor‐kappa B signaling. Cancer Res. 2008;68(20):8564–8572. 18922932 10.1158/0008-5472.CAN-08-2232 PMC3149885

[37] Yun JM , Afaq F , Khan N , Mukhtar H. Delphinidin, an anthocyanidin in pigmented fruits and vegetables, induces apoptosis and cell cycle arrest in human colon cancer HCT116 cells. Mol Carcinog. 2009;48(3):260–270. 18729103 10.1002/mc.20477 PMC2946888

[38] Mehlen P , Puisieux A. Metastasis: a question of life or death. Nat Rev Cancer. 2006;6(6):449–458. 16723991 10.1038/nrc1886

[39] Brooks SA , Lomax‐Browne HJ , Carter TM , Kinch CE , Hall DM. Molecular interactions in cancer cell metastasis. Acta Histochem. 2010;112(1):3–25. 19162308 10.1016/j.acthis.2008.11.022

[40] Gupta GP , Massague J. Cancer metastasis: building a framework. Cell. 2006;127(4):679–695. 17110329 10.1016/j.cell.2006.11.001

[41] Chaffer CL , Weinberg RA. A perspective on cancer cell metastasis. Science. 2011;331(6024):1559–1564. 21436443 10.1126/science.1203543

[42] Longhi A , Errani C , De Paolis M , Mercuri M , Bacci G. Primary bone osteosarcoma in the pediatric age: state of the art. Cancer Treat Rev. 2006;32(6):423–436. 16860938 10.1016/j.ctrv.2006.05.005

[43] Lim W , Jeong W , Song G. Delphinidin suppresses proliferation and migration of human ovarian clear cell carcinoma cells through blocking AKT and ERK1/2 MAPK signaling pathways. Mol Cell Endocrinol. 2016;422(C):172–181. 26704080 10.1016/j.mce.2015.12.013

[44] Ziegler U , Groscurth P. Morphological features of cell death. News Physiol Sci. 2004;19:124–128. 15143207 10.1152/nips.01519.2004

[45] Ramesh E , Alshatwi AA. Naringin induces death receptor and mitochondria‐mediated apoptosis in human cervical cancer (SiHa) cells. Food Chem Toxicol. 2013;51:97–105. 22847135 10.1016/j.fct.2012.07.033

[46] Fulda S , Debatin KM. Extrinsic versus intrinsic apoptosis pathways in anticancer chemotherapy. Oncogene. 2006;25(34):4798–4811. 16892092 10.1038/sj.onc.1209608

[47] Wang X. The expanding role of mitochondria in apoptosis. Genes Dev. 2001;15(22):2922–2933. 11711427

[48] Kroemer G , Galluzzi L , Brenner C. Mitochondrial membrane permeabilization in cell death. Physiol Rev. 2007;87(1):99–163. 17237344 10.1152/physrev.00013.2006

[49] Kuwana T , Newmeyer DD. Bcl‐2‐family proteins and the role of mitochondria in apoptosis. Curr Opin Cell Biol. 2003;15(6):691–699. 14644193 10.1016/j.ceb.2003.10.004

[50] Tse JC , Kalluri R. Mechanisms of metastasis: epithelial‐to‐mesenchymal transition and contribution of tumor microenvironment. J Cell Biochem. 2007;101(4):816–829. 17243120 10.1002/jcb.21215

[51] Krisanaprakornkit S , Iamaroon A. Epithelial‐mesenchymal transition in oral squamous cell carcinoma. ISRN Oncol. 2012;2012:681469 22548191 10.5402/2012/681469 PMC3324906

[52] Nieto MA. The snail superfamily of zinc‐finger transcription factors. Nat Rev Mol Cell Biol. 2002;3(3):155–166. 11994736 10.1038/nrm757

[53] Knight RD , Shimeld SM. Identification of conserved C2H2 zinc‐finger gene families in the Bilateria. Genome Biol. 2001;2(5):research0016–research0011. 11387037 10.1186/gb-2001-2-5-research0016 PMC32188

[54] Kataoka H , Murayama T , Yokode M , et al. A novel Snail‐related transcription factor Smuc regulates basic helix–loop–helix transcription factor activities via specific E‐box motifs. Nucleic Acids Res. 2000;28(2):626–633. 10606664 10.1093/nar/28.2.626 PMC102498

[55] Kalluri R , Weinberg RA. The basics of epithelial‐mesenchymal transition. J Clin Investig. 2009;119(6):1420 19487818 10.1172/JCI39104 PMC2689101

[56] Micalizzi DS , Farabaugh SM , Ford HL. Epithelial‐mesenchymal transition in cancer: parallels between normal development and tumor progression. J Mammary Gland Biol Neoplasia. 2010;15(2):117–134. 20490631 10.1007/s10911-010-9178-9 PMC2886089

[57] Haslehurst AM , Koti M , Dharsee M , et al. EMT transcription factors snail and slug directly contribute to cisplatin resistance in ovarian cancer. BMC Cancer. 2012;12(1):91 22429801 10.1186/1471-2407-12-91 PMC3342883

[58] Peinado H , Portillo F , Cano A. Transcriptional regulation of cadherins during development and carcinogenesis. Int J Dev Biol. 2004;48(5–6):365–375. 15349812 10.1387/ijdb.041794hp

[59] Batlle E , Sancho E , Franci C , et al. The transcription factor Snail is a repressor of E‐cadherin gene expression in epithelial tumour cells. Nat Cell Biol. 2000;2(2):84–89. 10655587 10.1038/35000034

[60] Cano A , Perez‐Moreno MA , Rodrigo I , et al. The transcription factor Snail controls epithelial‐mesenchymal transitions by repressing E‐cadherin expression. Nat Cell Biol. 2000;2(2):76–83. 10655586 10.1038/35000025

[61] Ye Q , Cai W , Zheng Y , Evers BM , She QB. ERK and AKT signaling cooperate to translationally regulate survivin expression for metastatic progression of colorectal cancer. Oncogene. 2014;33(14):1828–1839. 23624914 10.1038/onc.2013.122 PMC3966979

[62] Dhanasekaran DN , Reddy EP. JNK signaling in apoptosis. Oncogene. 2008;27(48):6245–6251. 18931691 10.1038/onc.2008.301 PMC3063296

[63] Wagner EF , Nebreda AR. Signal integration by JNK and p38 MAPK pathways in cancer development. Nat Rev Cancer. 2009;9(8):537–549. 19629069 10.1038/nrc2694

[64] del Barco Barrantes I , Nebreda AR. Roles of p38 MAPKs in invasion and metastasis. Biochem Soc Trans. 2012;40(1):79–84. 22260669 10.1042/BST20110676

